# Intakes of fruits and vegetables, carotenoids and vitamins A, E, C in relation to the risk of bladder cancer in the ATBC cohort study

**DOI:** 10.1038/sj.bjc.6600604

**Published:** 2002-10-21

**Authors:** D S Michaud, P Pietinen, P R Taylor, M Virtanen, J Virtamo, D Albanes

**Affiliations:** Nutritional Epidemiology Branch, Division of Cancer Epidemiology and Genetics, National Cancer Institute, Rockville, MD 20852, USA; Department of Epidemiology and Health Promotion, National Public Health Institute, Helsinki, Finland; Center for Cancer Research, National Cancer Institute, Rockville, Maryland, USA

**Keywords:** vitamins, carotenoids, fruit, vegetables, bladder cancer, cohort studies, epidemiology

## Abstract

We examined the relation between dietary fruit and vegetables, carotenoids and vitamin intakes and the risk of bladder cancer among male smokers in a prospective cohort study. Over a median of 11 years, we followed 27 111 male smokers aged 50–69 years who were initially enrolled in the Alpha-Tocopherol Beta-Carotene Cancer Prevention Study. During this period, 344 men developed bladder cancer. All of these men had completed a 276-food item dietary questionnaire at baseline. Cox proportional hazards models were used to estimate the relative risks and 95% confidence intervals and to simultaneously adjust for age, smoking history, energy intake and intervention group. Consumption of fruits and vegetables was not associated with the risk of bladder cancer (relative risk=1.28; 95% confidence intervals CI: 0.89–1.84, for highest *vs* lowest quintile). Similarly, no associations were observed for groups of fruits or vegetables (berries and cruciferous vegetables), or for specific fruits and vegetables. Dietary intakes of alpha-carotene, beta-carotene, lycopene, lutein/zeaxanthin, beta-cryptoxanthin, vitamins A, E, and C, and folate were not related to the risk of bladder cancer. These findings suggest that fruit and vegetable intakes are not likely to be associated with bladder cancer risk. However, these results may not be generalisable to non-smokers.

*British Journal of Cancer* (2002) **87**, 960–965. doi:10.1038/sj.bjc.6600604 www.bjcancer.com

© 2002 Cancer Research UK

## 

In a recent meta-analysis of diet and bladder cancer, a relative risk of 1.40 (95% confidence interval=1.08–1.83) was reported for a diet low in fruit intake (Steinmaus *et al*, 2000). Inverse associations were observed for total fruit consumption in four cohort studies (Mills *et al*, 1991; Shibata *et al*, 1992; Chyou *et al*, 1993; Nagano *et al*, 2000), but these studies had small numbers of bladder cancer (ranging from 52 to 106), and in only one was the difference significant (Nagano *et al*, 2000). In two other large prospective studies, no associations were observed for intakes of total fruits or vegetables (Michaud *et al*, 1999; Zeegers *et al*, 2001b).

Most observational studies have found no association of intakes of vitamins A and C with bladder cancer risk (World Cancer Research Fund & American Institute for Cancer Research, 1997a; Steinmaus *et al*, 2000). To date, only one case–control study has reported a significant inverse association with total dietary and supplemental vitamin C intake (Bruemmer *et al*, 1996). Three studies (Shibata *et al*, 1992; Bruemmer *et al*, 1996; Michaud *et al*, 2000) have observed inverse associations with supplement vitamin E (the first was not statistically significant). No associations were observed for intakes of specific carotenoids and risk of bladder cancer in two studies (Michaud *et al*, 2000; Zeegers *et al*, 2001a).

To clarify these questions among smokers, we examined these exposures in the Alpha-Tocopherol, Beta-Carotene Cancer Prevention (ATBC) Study.

## SUBJECTS AND METHODS

### Study cohort

The Alpha-Tocopherol, Beta-Carotene Cancer Prevention (ATBC) Study was established between 1985 and 1988 when 29 133 male smokers aged 50–69 years living in southwestern Finland agreed to participate and were randomised into the trial. The original trial, testing the effect of alpha-tocopherol and beta-carotene supplemental intakes on lung and other cancer risk using a 2×2 factorial design, ended in April 1993. Since then, all men in the trial have been followed-up for disease endpoints or death through the Finnish Cancer Registry and the Registry of Causes of Death. At baseline, men were excluded from the trial if they smoked less than five cigarettes per day, had prior cancer, a serious disease limiting long-term participation, or if they were users of vitamins E, A or beta-carotene supplements in excess of predefined doses (>20 mg per day of vitamin E, >20 000 IU per day of vitamin A, or >6 mg per day of beta-carotene). The rationale, methods, participation characteristics, compliance, and main results of the ATBC study are described in detail elsewhere (The Alpha-Tocopherol Beta-Carotene Cancer Prevention Study Group, 1994a,b). The study was approved by the institutional review boards of both the National Public Health Institute in Finland and the National Cancer Institute in the US.

Data on health status, smoking, height, weight and other characteristics were obtained at the time of entry into the trial. Men were excluded from the analyses if they did not complete the dietary questionnaire or if the dietary information was incomplete (*n*=2022), leaving a total of 27 111 men for analyses.

### Dietary assessment

The food use questionnaire included 276 food and beverage items commonly consumed in Finland and used a colour picture book to guide the subjects with respect to portion sizes. Participants were asked to report their average intake and portion size for each food over the past 12 months. Nutrient intakes were calculated by using the food composition database of the National Public Health Institute in Finland. Data on dietary carotenoid and retinol intakes were based primarily on food composition tables developed from HPLC analyses of Finnish foods (Heinonen *et al*, 1988a,b, 1989a,b; Ollilainen *et al*, 1989).

Over 45 items were aimed at capturing fruit and vegetable consumption, including seasonal differences for numerous fruits. In addition to combining all fruits (including berries) and vegetables for analysis, we created two other groups: cruciferous vegetables (broccoli, cauliflower, cabbage, Brussel sprouts, and rutabaga), and berries (strawberries, blueberries, black currants, red currants, raspberries, gooseberries, cloudberries, lingonberries). Carrots were the only frequently consumed ‘yellow/orange vegetables’ and citrus fruits were asked as one question (oranges, mandarins, grapefruit).

The questionnaire used for this study was tested for reproducibility and validity among 311 men selected to represent participants in the ATBC Study (Pietinen *et al*, 1988). In the validation study, 190 men completed 24 days of food records (12 records for two consecutive days each) distributed evenly over a 6-month period, and two food use questionnaires (one before and one after records). Correlations for all nutrients (corrected for attenuation) ranged between 0.50 for selenium to 0.86 for starch, and were 0.55, 0.70 and 0.76 for vitamins A, C, and E, respectively (Pietinen *et al*, 1988).

### Ascertainment of bladder cancer cases

All cases of bladder cancer were identified through the Finnish Cancer Registry and the Register of Causes of Death from baseline to the end of December 1998, providing complete ascertainment of cases (Kyllonen *et al*, 1987). Medical records were reviewed by one or two study physicians, and only cases with confirmed incident bladder cancer were included (ICD-9 code 188 and 233.7) (Services, 2001). Cancers of the renal pelvis, ureter and urethra (ICD-9 codes 189.1, 189.2 and 189.3) (Services, 2001) were not included in the present analyses. Over a median follow-up of 11 years, 344 men were diagnosed with bladder cancer among those who were eligible for the analyses in this study.

### Statistical analysis

We calculated person-years of observation for each participant from the date of randomisation to the date of bladder cancer diagnosis, death, or December 31, 1998, whichever came first. Men were categorised by quintiles (or categories) of dietary intakes using the baseline food use questionnaire. Nutrient analyses (carotenoids and vitamins) were adjusted for total energy intake using the residual method (Willett, 1990). We used Cox proportional hazards models to estimate risk and simultaneously adjust for age (continuous), duration of cigarette smoking (continuous), cigarette dose (continuous), and intervention assignment. In addition, we included total energy (quintiles) when modelling the fruit and vegetable items. Additional adjustment for potential confounders, including quintiles of fluid intake (from coffee, milk, juice, etc, but not including non-bottled water as it was not assessed), education, body mass index (BMI), area of residence, and smoking inhalation, did not change the associations of the nutrients and foods presented in this paper. Test for trends were conducted by using the median values for each quintile of food or nutrient and modelling them as a continuous variable. All *P* values are two-sided.

## RESULTS

During a median follow-up time of 11 years, 344 cases of bladder cancer were diagnosed in the cohort. On average, men with a high fruit and vegetable intake had smoked fewer cigarettes and fewer years, had attained higher levels of education, and were less likely to live in small towns/rural areas than men who consumed low amounts of fruits and vegetables (Table 1

**Table 1:**
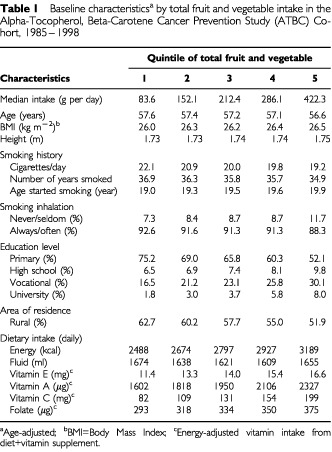
Baseline characteristics^a^ by total fruit and vegetable intake in the Alpha-Tocopherol, Beta-Carotene Cancer Prevention Study (ATBC) Cohort, 1985–1998

). Intakes of total energy, folate, vitamins A, C, and E were all higher among frequent consumers of fruits and vegetables.

Consumption of fruits and vegetables was not associated with the risk of bladder cancer in this cohort of male smokers (Table 2

**Table 2:**
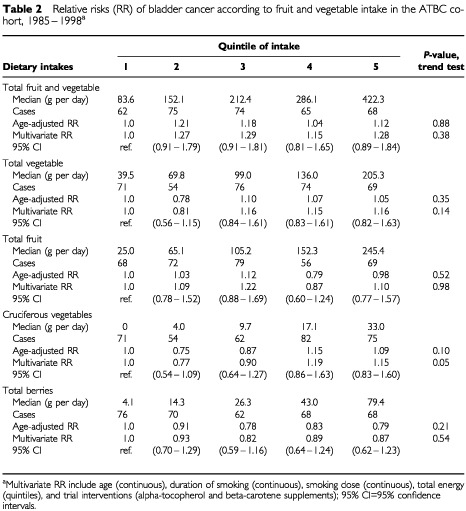
Relative risks (RR) of bladder cancer according to fruit and vegetable intake in the ATBC cohort, 1985–1998^a^

). Controlling for smoking history, trial group, and energy intake did not change the relative risks substantially and there was no indication of an inverse association for either total fruit, or total vegetable intake. Similarly, we observed no relation between intakes of cruciferous vegetables or total berries and the risk of bladder cancer (Table 2). Results for total fruit and vegetable intakes, as well as those for the different groupings, were similar across strata of smoking duration (data not shown).

We selected frequently consumed vegetable items and examined these individually (carrots, peas, tomatoes, and cauliflower). None of these vegetable items were associated with the risk of bladder cancer (data not shown). Few people reported eating broccoli; however, among those who reported consuming any broccoli during the year, we observed a statistically nonsignificant relative risk of 0.78 (95% CI=0.50–1.23) compared to those who never reported eating broccoli.

Intakes of α-carotene, β-carotene, lutein/zeaxanthin, lycopene, and β-cryptoxanthin were not related to the risk of bladder cancer in both age-adjusted and multivariate analyses (Table 3

**Table 3:**
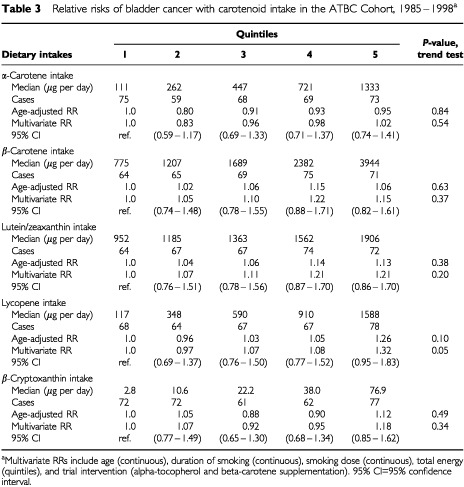
Relative risks of bladder cancer with carotenoid intake in the ATBC Cohort, 1985–1998^a^

). Findings for the individual carotenoid intakes remained null within strata of smoking duration (⩽35, 36–45, >45 years of smoking), which was the strongest predictor of bladder cancer risk in this cohort (data not shown).

Dietary intakes of vitamins A, C, E and folate were not associated with the risk of bladder cancer (Table 4

**Table 4:**
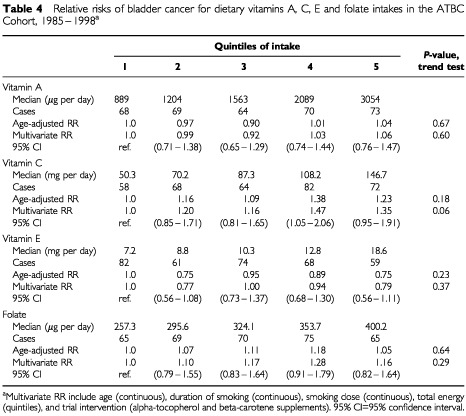
Relative risks of bladder cancer for dietary vitamins A, C, E and folate intakes in the ATBC Cohort, 1985–1998^a^

). Vitamin supplements (mostly from multivitamins) reported on the baseline questionnaire did not contribute substantially to total vitamin intake as fewer than 15% of the men in this cohort reported taking some form of vitamin supplement prior to the study intervention. Adding vitamin supplements to the dietary intakes did not result in substantial changes in the relative risk estimates (data not shown). Supplemental intakes of vitamins A, C, E, or folate were not related to the risk of bladder cancer (data not shown).

We stratified the vitamin analyses by the four groups of the ATBC trial (alpha-tocopherol and beta-carotene) to examine whether associations for vitamin A, E and C intakes were modified by supplementation of beta-carotene and vitamin E. A suggestive inverse association was observed for total vitamin E intake and bladder cancer risk in the placebo group (Table 5

**Table 5:**
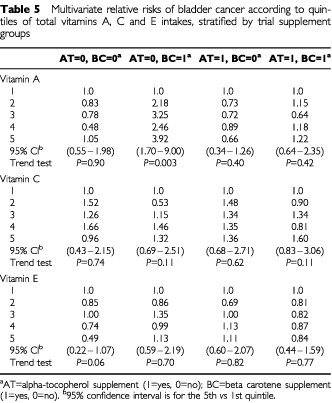
Multivariate relative risks of bladder cancer according to quintiles of total vitamins A, C and E intakes, stratified by trial supplement groups

). A statistically significant elevation in risk of bladder cancer was observed for high *vs* low levels of vitamin A intake at baseline, but this was only observed in the trial arm taking beta-carotene supplements alone and not in the other three groups.

## DISCUSSION

No associations were observed in this cohort of male smokers between the consumption of fruits and vegetables and the risk of bladder cancer. Groupings by different types of fruits and vegetables (e.g., berries, cruciferous vegetables) gave similar null results. Dietary intakes of alpha-carotene, beta-carotene, lycopene, lutein/zeathanthin, and beta-cryptoxanthin were not related to bladder cancer risk. Neither dietary nor total intakes (including supplement use) of vitamins A, E, C and folic acid were associated with bladder cancer risk.

The majority of studies that have examined dietary factors and bladder cancer risk have been case–control studies. In a recent meta-analysis of published data (through 1998) on six dietary factors and bladder cancer risk the authors concluded that diets low in fruits, and to a lesser extent those low in vegetables, were associated with an increased risk of bladder cancer (Steinmaus *et al*, 2000). In this meta-analysis, the authors did not stratify the results by smoking status. When examined individually, however, findings have been inconsistent across studies. This is perhaps not surprising given the diversity of populations studied and methodological concerns, including small numbers in five prospective studies, varying numbers of questions on consumption of fruits and vegetables (between 2 and 44 items), and the possibility of recall bias and other biases in case–control studies.

The lack of associations in the present study raises some methodological issues. Nondifferential misclassification from measurement error can bias associations towards the null. In this study, a quantitative food use questionnaire was administered to participants and assistance was offered to those who had difficulty understanding the questions. While we cannot exclude the possibility that the lack of association may be due to misclassification, it is unlikely that there would be more misclassification in this study than in previous studies that have observed inverse associations using similar dietary assessments (Mills *et al*, 1991; Shibata *et al*, 1992; Bruemmer *et al*, 1996). Confounding by occupational exposures may have occurred if men in high-risk occupations consumed more fruits and vegetables than the rest of the population.

A lack of association may also arise if the range of intake for the exposure of interest is not sufficiently large. The number of fruit and vegetable items in the questionnaire in this study far surpasses most previous studies on this topic (over 45 items for individual fruits and vegetables) and the range for total fruits and vegetables is greater or similar to those reported in previous studies (Zeegers *et al*, 2001b). Although this holds true for total fruits and vegetables, some individual vegetables, including broccoli, were eaten infrequently in this population, possibly explaining the lack of association with cruciferous vegetables.

Another possible explanation for the lack of association observed for cruciferous vegetable intake in this study may be cigarette smoking. In the Health Professionals Follow-Up Study (Michaud *et al*, 1999), cruciferous vegetable intake was inversely related to bladder cancer risk, but after stratifying by smoking status the inverse association was only apparent among nonsmokers. It may be that smokers do not benefit from the effects of consuming a diet high in cruciferous vegetables. Cruciferous vegetables are high in glucosinolates, which are hydrolysed to isothiocyanates in the body (Zhang and Talalay, 1994). These compounds can enhance the activity of detoxifying enzymes such as glutathione S-transferase (Zhang and Talalay, 1994); however, these enzymes are not involved in the detoxification of aromatic amines, which appear to be the most important bladder carcinogens found in cigarettes (Vineis and Pirastu, 1997). In the Netherlands Cohort Study, the authors did not indicate whether the inverse association between cauliflower and bladder cancer risk was modified by smoking status (Zeegers *et al*, 2001b).

To our knowledge, only three studies (Garcia *et al*, 1999; Michaud *et al*, 1999; Zeegers *et al*, 2001a) have published findings for the relation between individual carotenoid intakes and bladder cancer risk. No associations were observed for alpha-carotene, beta-carotene, lycopene or lutein intakes in these publications, but an inverse association was reported for beta-cryptoxanthin in the Netherlands Cohort Study, which was particularly strong among heavy smokers (Zeegers *et al*, 2001a). Carotenoids have many properties that may be important in the prevention of carcinogenesis, antioxidation having been the primary focus in many studies. Recent research found that carotenoids have other pertinent properties, including antimutagenic and anticlastogenic effects (Rauscher *et al*, 1998), inhibitory effects on Epstein-Barr virus activation activity of a tumour promoter (Tsushima *et al*, 1995), and cytoprotective effects (Javor *et al*, 1983).

The lack of an association for vitamin E intake does not exclude the possibility that an effect for vitamin E exists among nonsmokers or persons with 10 or more years of supplement use. In a cohort of male professionals, an inverse association was observed for vitamin E supplement use and bladder cancer risk, and the lowest relative risk was seen among men with 10 or more years of vitamin E supplement use (Michaud *et al*, 2000). Furthermore, the association was not observed among smokers, although there was limited power for this analysis. Vitamin E supplement use has been inversely related to bladder cancer risk in two other case–control studies with supplement data (Shibata *et al*, 1992; Bruemmer *et al*, 1996) (although only the latter was statistically significant). In addition, although statistically insignificant, our results are consistent with a small decrease in risk among high vitamin E consumers. Although supplementation with vitamin E for 5–8 years had no effect on urothelial cancer risk (Virtamo *et al*, 2000), a longer exposure period may be necessary for high levels of vitamin E to affect the risk of bladder cancer.

While early studies indicated that vitamin A intake may be related to bladder cancer risk (Kolonel *et al*, 1985; Mettlin and Graham, 1979), numerous other studies have not confirmed these findings, and retinol intake was not related to risk of bladder cancer in a meta-analysis (Steinmaus *et al*, 2000). Our findings are consistent with this, as well as for a null finding for vitamin C intake (World Cancer Research Fund & American Institute for Cancer Research, 1997b).

We did not observe an inverse association for total fruit and vegetable intakes and bladder cancer risk in this cohort of male smokers. In two previous cohorts, inverse associations were observed for cruciferous vegetables, however, because broccoli and cauliflower were not frequently consumed in this population we had limited power to examine these specific vegetables. Dietary and supplemental vitamins A, E, and C, and carotenoids were not related to bladder cancer risk. Based on these findings, it is unlikely that total fruits and vegetables play a major role in bladder cancer prevention among smokers. Future studies should focus on the association between intakes of fruits and vegetables and bladder cancer risk among nonsmokers.

## References

[bib1] BruemmerBWhiteEVaughanTLCheneyCL1996 Nutrient intake in relation to bladder cancer among middle-aged men and women Am J Epidemiol 144 485495 8781464 10.1093/oxfordjournals.aje.a008955

[bib2] ChyouP-HNomuraAMYStemmermannGN1993 A prospective study of diet, smoking, and lower urinary tract cancer Ann Epidemiol 3 211216 8275191 10.1016/1047-2797(93)90021-u

[bib3] GarciaRGonzalezCAAgudoARiboliE1999 High intake of specific carotenoids and flavonoids does not reduce the risk of bladder cancer Nutr Cancer 35 212214 10693178 10.1207/S15327914NC352_18

[bib4] HeinonenMIOllilainenVLinkolaEKVaroPTKoivistoinenPE1988a Carotenoids and retinoids in Finnish foods: dietary fats J Food Comp Anal 1 334340

[bib5] HeinonenMIOllilainenVLinkolaEKVaroPTKoivistoinenPE1988b Carotenoids and retinoids in Finnish foods: meat and meat products J Food Comp Anal 1 178188

[bib6] HeinonenMIOllilainenVLinkolaEKVaroPTKoivistoinenPE1989a Carotenoids and retinoids in Finnish foods: cereal and bakery products Cereal Chem 66 270273

[bib7] HeinonenMIOllilainenVLinkolaEKVaroPTKoivistoinenPE1989b Carotenoids in Finnish foods: vegetables, fruits, and berries J Agric Food Chem 37 655659

[bib8] JavorTBataMLovaszLMoronFNagyLPattyISzabolcsJTarnokFTothGMozsikG1983 Gastric cytoprotective effects of vitamin A and other carotenoids Int J Tissue React 5 289296 6654625

[bib9] KolonelLNHindsMWNomuraAMYHankinJHLeeJ1985 Relationship of dietary vitamin A and ascorbic acid intake to the risk for cancers of the lung, bladder, and prostate in Hawaii Natl Cancer Inst Monogr 69 137142 3834323

[bib10] KyllonenLETeppoLLehtonenM1987 Completeness and accuracy of registration of colorectal cancer in Finland Ann Chir Gynaecol 76 185190 3434988

[bib11] MettlinCGrahamS1979 Dietary risk factors in human bladder cancer Am J Epidemiol 110 255263 582494 10.1093/oxfordjournals.aje.a112810

[bib12] MichaudDSpiegelmanDClintonSRimmEWillettWGiovannucciE1999 Fruit and vegetable intake and incidence of bladder cancer in a male prospective cohort J Natl Cancer Inst 91 605613 10203279 10.1093/jnci/91.7.605

[bib13] MichaudDSSpiegelmanDClintonSKRimmEBWillettWCGiovannucciE2000 Prospective study of dietary supplements, macronutrients, micronutrients, and risk of bladder cancer in US men Am J Epidemiol 152 11451153 11130620 10.1093/aje/152.12.1145

[bib14] MillsPKBeesonWLPhillipsRLFraserGE1991 Bladder cancer in a low risk population: results from the Adventist Health Study Am J Epidemiol 133 230239 2000840 10.1093/oxfordjournals.aje.a115867

[bib15] NaganoJKonoSPrestonDLMoriwakiHSharpGBKoyamaKMabuchiK2000 Bladder-cancer incidence in relation to vegetable and fruit consumption: a prospective study of atomic-bomb survivors Int J Cancer 86 132138 10728607 10.1002/(sici)1097-0215(20000401)86:1<132::aid-ijc21>3.0.co;2-m

[bib16] OllilainenVHeinonenMILinkolaEKVaroPTKoivistoinenPE1989 Carotenoids and retinoids in Finnish foods: dairy products and eggs J Dairy Sci 72 22572265 2592640 10.3168/jds.S0022-0302(89)79356-5

[bib17] PietinenPHartmanAMHaapaERasanenLHaapakoskiJPalmgrenJAlbanesDVirtamoJHuttunenJK1988 Reproducibility and validity of dietary assessment instruments. I. A self-administered food use questionnaire with a portion size picture booklet Am J Epidemiol 128 655666 2458036 10.1093/oxfordjournals.aje.a115013

[bib18] RauscherREdenharderRPlattKL1998 In vitro antimutagenic and in vivo anticlastogenic effects of carotenoids and solvent extracts from fruits and vegetables rich in carotenoids Mutat Res 413 129142 9639691 10.1016/s1383-5718(98)00017-5

[bib19] Services, U.D.o.H.a.H2001 International Classification of Diseases, 9th Revision, Clinical Modification.US Public Health Service: Washington, DC

[bib20] ShibataAPaganini-HillARossRKHendersonBE1992 Intake of vegetables, fruits, beta-carotene, vitamin C and vitamin supplements and cancer incidence among the elderly: a prospective study Br J Cancer 66 673679 1419605 10.1038/bjc.1992.336 PMC1977409

[bib21] SteinmausCMNunezSSmithAH2000 Diet and bladder cancer: a meta-analysis of six dietary variables Am J Epidemiol 151 693702 10752797 10.1093/oxfordjournals.aje.a010264

[bib22] The Alpha-Tocopherol Beta-Carotene Cancer Prevention Study Group1994a The Alpha-Tocopherol, Beta-Carotene Lung Cancer Prevention Study: Design, methods, participant characteristics, and compliance Ann Epidemiol 4 110 8205268 10.1016/1047-2797(94)90036-1

[bib23] The Alpha-Tocopherol Beta-Carotene Cancer Prevention Study Group1994b The effect of vitamin E and beta carotene on the incidence of lung cancer and other cancers in male smokers N Engl J Med 330 10291035 8127329 10.1056/NEJM199404143301501

[bib24] TsushimaMMaokaTKatsuyamaMKozukaMMatsunoTTokudaHNishinoHIwashimaA1995 Inhibitory effect of natural carotenoids on Epstein-Barr virus activation activity of a tumor promoter in Raji cells. A screening study for anti-tumor promoters Biol Pharm Bull 18 227233 7742789 10.1248/bpb.18.227

[bib25] VineisPPirastuR1997 Aromatic amines and cancer Cancer Causes Control 8 346355 9498898 10.1023/a:1018453104303

[bib26] VirtamoJEdwardsBKVirtanenMTaylorPRMalilaNAlbanesDHuttunenJKHartmanAMHietanenPMaenpaaHKossLNordlingSHeinonenOP2000 Effects of supplemental alpha-tocopherol and beta-carotene on urinary tract cancer: incidence and mortality in a controlled trial (Finland) Cancer Causes Control 11 933939 11142528 10.1023/a:1026546803917

[bib27] WillettWC1990 Nutritional Epidemiology.Oxford University Press: New York, NY

[bib28] World Cancer Research Fund & American Institute for Cancer Research1997a Food, nutrition and the prevention of cancer: a global perspective.American Institute for Cancer Research: Washington, DC 10.1016/s0899-9007(99)00021-0 10378216

[bib29] World Cancer Research Fund & American Institute for Cancer Research1997b Food, Nutrition and the Prevention of Cancer: a Global Perspective.American Institute for Cancer Research: Washington, DC 10.1016/s0899-9007(99)00021-0 10378216

[bib30] ZeegersMPGoldbohmRABrandtPA2001a Are retinol, vitamin C, vitamin E, folate and carotenoids intake associated with bladder cancer risk? Results from the Netherlands Cohort Study Br J Cancer 85 977983 11592769 10.1054/bjoc.2001.1968 PMC2375109

[bib31] ZeegersMPGoldbohmRAvan Den BrandtPA2001b Consumption of vegetables and fruits and urothelial cancer incidence: a prospective study Cancer Epidemiol Biomarkers Prev 10 11211128 11700259

[bib32] ZhangYTalalayP1994 Anticarcinogenic activities of organic isothiocyanates: chemistry and mechanisms Cancer Res 54 1976s1981s 8137323

